# Comparing vector and human surveillance strategies to detect arbovirus transmission: A simulation study for Zika virus detection in Puerto Rico

**DOI:** 10.1371/journal.pntd.0007988

**Published:** 2019-12-26

**Authors:** Zachary J. Madewell, Ryan R. Hemme, Laura Adams, Roberto Barrera, Stephen H. Waterman, Michael A. Johansson

**Affiliations:** 1 Centro de Estudios en Salud, Universidad del Valle de Guatemala, Guatemala City, Guatemala; 2 University of California, San Diego/San Diego State University, PhD Program in Public Health (Epidemiology), La Jolla, California, United States of America; 3 Dengue Branch, Centers for Disease Control and Prevention (CDC), San Juan, Puerto Rico, United States of America; International Centre for Genetic Engineering and Biotechnology, INDIA

## Abstract

**Background:**

Detecting and monitoring the transmission of arboviruses such as Zika virus (ZIKV), dengue virus, and chikungunya virus is critical for prevention and control activities. Previous work has compared the ability of different human-focused surveillance strategies to detect ZIKV transmission in U.S. counties where no known transmission had occurred, but whether virological surveillance in mosquitoes could represent an effective surveillance system is unclear.

**Objectives:**

We leveraged a unique set of data from human and virological surveillance in *Ae*. *aegypti* during the 2016 ZIKV epidemic in Caguas, Puerto Rico, to compare alternative strategies for detecting and monitoring ZIKV activity.

**Methods:**

We developed a simulation model for mosquito and human surveillance strategies and simulated different transmission scenarios with varying infection rates and mosquito trap densities. We then calculated the expected weekly number of detected infections, the probability of detecting transmission, and the number of tests needed and compared the simulations with observed data from Caguas.

**Results:**

In simulated high transmission scenarios (1 infection per 1,000 people per week), the models demonstrated that both approaches had estimated probabilities of detection of greater than 78%. In simulated low incidence scenarios, vector surveillance had higher sensitivity than human surveillance and sensitivity increased with more traps, more trapping effort, and testing. In contrast, the actual data from Caguas indicated that human virological surveillance was more sensitive than vector virological surveillance during periods of both high and low transmission.

**Conclusion:**

In scenarios where human surveillance is not possible or when transmission intensity is very low, virological surveillance in *Ae*. *aegypti* may be able to detect and monitor ZIKV epidemic activity. However, surveillance for humans seeking care for Zika-like symptoms likely provides an equivalent or more sensitive indicator of transmission intensity in most circumstances.

## Author summary

Control of Zika virus and other mosquito-borne viruses is dependent on timely and accurate detection of viral activity. A previous assessment of surveillance strategies for U.S. counties where no known transmission had occurred, found that testing people seeking medical care with signs of Zika virus was a more effective strategy than testing blood donors or pregnant women. An alternative strategy to testing humans is to test the mosquitoes directly for viruses. In this study, we used data from human and mosquito surveillances during the Zika epidemic of 2016 in Caguas, Puerto Rico to compare these strategies for detecting and monitoring Zika virus activity. Using simulation models for human and mosquito surveillance strategies, we examined different transmission scenarios with varying Zika virus infection rates. The results suggest that in high Zika virus transmission scenarios, both approaches effectively identified transmission. In low transmission scenarios, routine mosquito surveillance had the potential for increased sensitivity compared to human-based surveillance. However, resource availability may also be an important factor when considering the most effective approach.

## Introduction

First identified in Uganda in 1947, Zika virus (ZIKV) emerged in the Americas in 2015 [1, 2], with local transmission of ZIKV first reported in December, 2015, in Puerto Rico [3]. The emergence of ZIKV highlighted several challenges for arbovirus surveillance. Many infections do not result in apparent illness [4] and acutely ill individuals do not always seek care or receive confirmatory testing [5, 6]. Moreover, multiple surveillance strategies are possible (e.g. active and passive, vector-based and human); all have tradeoffs that may vary depending on the epidemiological situation. For example, a previous assessment of human-based surveillance systems to detect ZIKV transmission in U.S. counties found that despite low probabilities of care-seeking among ZIKV-infected individuals, testing symptomatic people seeking medical care was a more effective strategy than testing blood donors or all pregnant women [5]. Testing pregnant women was found to be a less efficient strategy because it requires more tests, has a much higher false positive rate, and has a lower probability of detection than testing only patients with two or more Zika symptoms.

Effective prevention and control of arboviruses such as ZIKV, dengue virus, and chikungunya virus is dependent on timely and accurate detection of elevated viral activity in the population. Surveillance systems detect virus circulation, track geographical spread, monitor epidemic progression, detect adverse health outcomes associated with infection, and guide response efforts [7, 8].

Although previous work suggested that testing clinical patients with at least two ZIKV symptoms was a relatively effective strategy for detecting ZIKV transmission in humans, the probability of detection was <25% even under optimal circumstances and incidence of one infection per 10,000 people per week in a population of 100,000 [5]. Moreover, none of the surveillance strategies evaluated could detect even 5% of all ZIKV infections.

An alternative to detecting arboviruses in humans is virological surveillance in mosquitoes, which involves capturing and testing host seeking female mosquitoes. For West Nile virus, which has a primary transmission cycle between infected *Culex* mosquitoes and birds, virological surveillance in mosquitoes can facilitate viral detection prior to human disease case identification [9, 10]. However, transmission of Zika, dengue, and chikungunya viruses occurs mainly between *Aedes* mosquitoes and human hosts. Even during outbreaks, detecting these viruses in *Ae*. *aegypti* is infrequent [11]. In this context, the possible tradeoffs between vector and human virological surveillance are unclear. Here, we leveraged a unique set of data from human and vector surveillance during the 2016 ZIKV epidemic in Caguas, Puerto Rico to compare strategies for detecting and monitoring arbovirus activity.

## Methods

### Ethics statement

This study includes frequencies of ZIKV cases in humans obtained from online weekly reports from the Department of Health, Puerto Rico. This is routine surveillance data that is publicly available and there is no protected health information. All data analyzed were anonymized.

### Study site

Caguas is located in the central mountain range of Puerto Rico, 32 km south of San Juan (18.23412° N, −66.0485° W). It has a population of approximately 130,000 people [12]. The municipality has a tropical climate with a mean annual temperature of 25.1°C and mean annual precipitation of 1,755 mm [13].

### Vector surveillance data

From October, 2016, to August, 2017, 360 sentinel autocidal gravid ovitraps (SAGO) were placed in eight clusters in Caguas encompassing approximately 80,000 residents [14, 15]. Within each cluster, fixed SAGOs were placed in outdoor areas of randomly selected households located at least 100 m from the adjacent clusters with 109–131 m between each trap. If the homeowner did not give consent or was not home, traps were placed in a location adjacent to the randomly selected site. Each cluster had 38–53 traps placed among approximately 3,000 built structures for an overall trap density of approximately 0.14 traps per building or one trap per 70.5 buildings. Traps were monitored on a weekly basis by field technicians, mosquitoes were sexed, and identified as *Ae*. *aegypti* in the field [14]. Female *Ae*. *aegypti* from individual SAGOs were pooled by week with 1–20 mosquitoes per pool and stored at −80°C until they were tested for viral RNA using the Trioplex Real-time RT-PCR assay [16]. ZIKV may be detected in *Ae*. *aegypti* held in traps at ambient temperatures [17]. We also simulated collections with higher and lower trap numbers.

### Human surveillance data

We collected human case data for Caguas from October 10, 2016–April 16, 2017, as reported by the Puerto Rico Department of Health (PRDH) from the passive arboviral disease surveillance system [18]. All symptomatic cases were reported to PRDH as suspected arbovirus infections based on clinical suspicion, with subsequent confirmatory laboratory testing. We assessed the number of ZIKV cases detected by RT-PCR (confirmed cases) compared with the total number of cases submitted for testing.

### Simulation model

We simulated surveillance for ZIKV-infected *Ae*. *aegypti* and humans considering the surveillance systems implemented in Caguas during the ZIKV epidemic. We developed simulation models to estimate ZIKV transmission and detection in humans and mosquitoes at varying transmission levels. Four surveillance strategies were assessed: mosquito trapping systems with 180, 360, and 720 traps; and human surveillance. We started by simulating population sizes of humans and vectors, the incidence of infection in both populations, and surveillance for infection in each population. We then compared the total number of detections and probability of detection between the strategies. These processes are described in detail below with key parameters in Table 1.

**Table 1:** Parameter assumptions for *Aedes aegypti* surveillance for Zika virus.

Parameter	Estimate	Uncertainty distribution	Source
Duration of human infectiousness	7 days (0.3 SD)	Gamma	[19]
Number of *Ae*. *aegypti* per person	2 (1.5 SD)	Gamma	[19–21]
Mosquito biting rate	0.63–0.76 bites per day	Uniform	[20, 22]
Probability of human-to-mosquito transmissibility	0.5 (0.1 SD)	Normal	[19, 20]
Number of *Ae*. *aegypti* per pool	5.17	Poisson	Caguas data
Sensitivity of RT-PCR Assay	85–100%	Uniform	[16, 23–25]
Specificity of RT-PCR Assay	99.99–100%	Uniform	[16, 23–25]

RT-PCR: reverse transcription polymerase chain reaction

#### Population sizes

We used a population of 100,000 people, slightly smaller than the total population of Caguas and larger than the population of the area covered by mosquito surveillance. The total number of mosquitoes, *N*_*M*_, was estimated from the density of *Ae*. *aegypti* (*r*) and human population size. We assumed there were 1 to 3 adult female *Ae*. *aegypti* per person, which we assumed to be gamma distributed [19–21].

#### Incidence of infection

We simulated scenarios with human incidence ranging from one infection per 100,000 per week to one infection per 1,000 per week. In these simulations, we used the incidence in humans to calculate the number of infected mosquitoes using an average number of mosquitoes infected by an infectious person (*R*_0*HM*_). *R*_0*HM*_ was calculated as the product of the density of mosquitoes (*r*), mosquito biting rate (*b*), probability of human-to-mosquito transmission given an infectious bite (*p*_*HM*_), and duration of infectiousness in humans (*d*): *R*_0*HM*_ = *rbp*_*HM*_*d* [19]. We estimated ranges and uncertainty distributions for each of these variables based on previous research. We assumed *b* = 0.63–0.76 bites per mosquito per day, which we approximated with a uniform distribution [22], and *p*_*HM*_ = 0.5 (SD = 0.1), which we approximated with a normal distribution [19, 20]. The duration of infectiousness in humans (*d*) was assumed to be similar to dengue, with a mean of 7 days (SD = 0.3) and approximated with a gamma distribution [19]. The expected number of ZIKV-infected female *Ae*. *aegypti* per week was estimated as the number of infectious humans multiplied by *R*_0*HM*_. The probability of a single female *Ae*. *aegypti* being infected when trapped was calculated as the expected number of ZIKV-infected female *Ae*. *aegypti* divided by the mosquito population size (*N*_*M*_).

#### Surveillance strategies

We modeled four different surveillance strategies. Three of the strategies used *Ae*. *aegypti* surveillance data at different trap densities: the number of traps equivalent to what was used in the study area of Caguas (360 traps), half as many traps (180 traps), and twice as many traps (720 traps). The fourth model used human surveillance for acutely ill patients with ZIKV symptoms. The four surveillance strategies were evaluated at different transmission densities, ranging from one to 1,000 human cases per 100,000 per week.

Human surveillance data was assumed to be similar to previously published estimates for identifying ZIKV cases from emergency departments in the continental United States [5]. Briefly, we assumed that 20–40% of infections result in symptoms, 10–50% of people with symptomatic infections seek care, 5–50% of those patients seek care in an emergency department, and 82.4–83.3% of those patients have at least two of the following ZIKV symptoms: arthralgia, conjunctivitis, fever, headache, or rash. Human surveillance in Puerto Rico differs from this simulation in at least two ways: reporting was not limited to emergency departments and reporting of suspect cases relies on clinical suspicion, not a specific clinical definition. We used the emergency department assumption and stricter case definition to approximate patients seeking immediate care for acute illness with clinical suspicion of ZIKV. For reported suspect ZIKV patients, we assumed all testing was performed with RT-PCR with sensitivity of 80–95% [26] and specificity of 99–100% [16].

For vector surveillance, we started each simulation with a specific number of traps and simulated the total numbers of mosquitoes and pools collected from those traps based on the Caguas collection data. The probability of a pool containing a positive mosquito was calculated as the product of the average number of mosquitoes collected per pool and the probability of an individual mosquito being infected (described above). Mosquito testing was performed with RT-PCR. Two studies demonstrated sensitivities of 88.7% [25] and 96.1% [27] for RT-PCR detection of ZIKV in mosquitoes. Based on those findings and studies of RT-PCR for detecting WNV in mosquitoes [23, 24], we assumed the test has a sensitivity of 85%-100% and a specificity of 99.9–100%, which we approximated with uniform distributions. ZIKV RNA is stable enough to allow detection for up to 30 days [28]. Because mosquitoes were collected and tested every 7 days, we assumed that all infected mosquitoes had detectable RNA [5].

#### Probability of detection

We first calculated the numbers of expected true and false positives from the human and mosquito surveillance strategies. For humans, the expected number of true positives (*E*(*P*)) was the product of the prevalence of infection, the probability of being tested in the surveillance system, and the sensitivity of the human RT-PCR assay. The expected number of false positives was the product of the probability of being tested in the surveillance system for people not infected with ZIKV and one minus the specificity of the human RT-PCR assay. The expected number of true positive mosquito pools (*E*(*P*)) was the product of the total number of pools, the probability of a pool containing an infected mosquito, and the sensitivity of the mosquito RT-PCR assay. Finally, the expected number of false positive pools was calculated as the product of the total number of pools, the probability of a pool not containing an infected mosquito, and one minus the specificity of the RT-PCR assay.

To estimate the probability of detection for each system we assumed that the number of true infections detected in mosquito pools or human patients was generated from a Poisson distribution with the means as the expected number of true positives as described above (*E*(*P*)). We therefore calculated the probability of detection, *p*_*detect*_, as *p*_*detect*_ = 1−*e*^−(*E*(*P*))^.

#### Estimating infections in human population

We fitted Bayesian negative binomial regression models to the simulation data to estimate the relationship between the number of positive pools or clinical infections and the total number of human infections (*I*_*H*_). We used linear models for both the mean parameter (*μ*) and the dispersion parameter (*ϕ*):

$$I_{H}∼\mathrm{NegativeBinomial}(\mu ,\phi )$$

$$\mu =\beta_{0}+\beta_{X}X$$

$$\phi =\beta_{0}^{\phi }+\beta_{X}^{\phi }X,$$

where *X* was the number of positive pools or the number of detected clinical infections.

To assess the relationship between our simulations and the observed data from Caguas, we first fitted these models to the simulation data in order to estimate the relationship between the number of infected humans or mosquito pools observed by the surveillance system and the number of underlying human infections in the population (130,000 for the human surveillance and 80,000 for the mosquito surveillance). We then used the fitted model to estimate the number of infections in the two populations based on the observed numbers of human cases and positive pools from the 2016–2017 surveillance data.

#### Software

For each surveillance strategy, we ran 10,000 Monte Carlo simulations. All analyses were performed in R 3.5.2 statistical software (R Development Core Team, Vienna, Austria). We used Stan to implement the Bayesian models. We used three chains with a burn-in of 1,000 samples and a further 1,000 samples collected from each chain with clear convergence and no significant autocorrelation.

## Results

**Fig 1:**
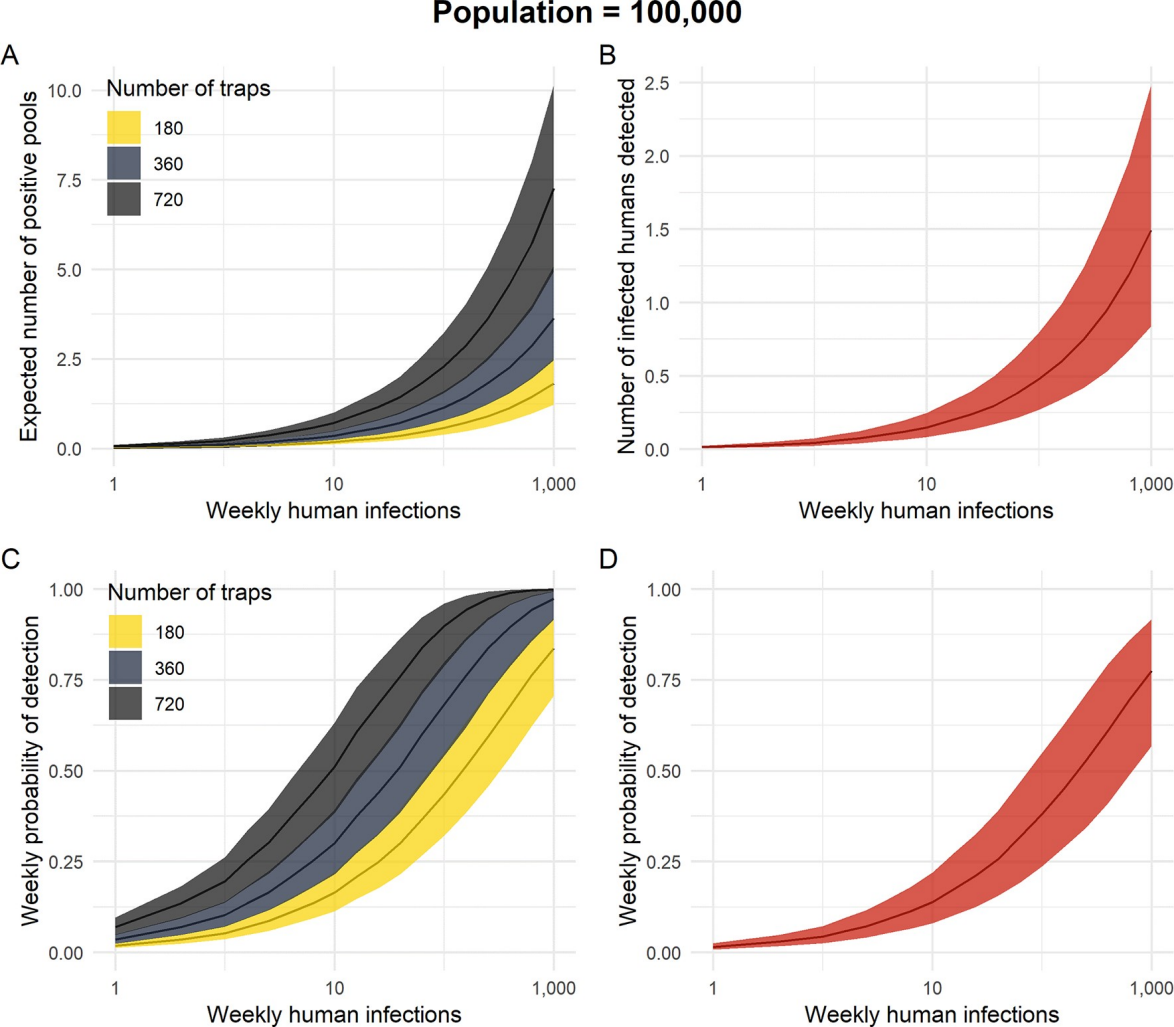
The total number of Zika virus infections detected and probability of detecting local transmission of Zika virus by testing *Aedes aegypti* females collected from CDC sentinel autocidal gravid ovitraps (SAGO) and emergency department patients with two or more Zika virus symptoms. Panel A shows the expected number of ZIKV-positive *Ae*. *aegypti* pools detected per week by testing pools of *Ae*. *aegypti* females trapped in the actual number of traps deployed in Caguas, Puerto Rico (360 SAGO traps: navy), half the number of traps as Caguas (180 SAGO traps: yellow), and twice the number of traps as Caguas (720 SAGO traps: black). Panel B shows the expected number of infected humans detected per week by testing patients in emergency departments showing two or more ZIKV symptoms (red). Panel C shows the weekly probability of detecting ZIKV by testing mosquito pools. Panel D shows the weekly probability of detecting ZIKV by testing symptomatic emergency department patients. The bands represent 50% uncertainty intervals.

We first analyzed the expected number of positive mosquito pools, human cases, and the probability of detecting infected vectors or humans for the four simulated surveillance strategies as described above. In a population of 100,000 and one infection per week, the expected number of positive mosquito pools or positive humans was essentially zero for all four systems (Fig 1A and 1B) (Table 2). Therefore, the probability of detecting a positive mosquito pool or infected human was very low, but highest (6.9%; 95% UI: 1.6%, 15.7%) for the mosquito trapping system with 720 traps and lowest for human surveillance (1.5%; 95% UI: 0.3%, 4.7%) (Fig 1C and 1D). When the incidence of infection increased to 10 infections per week, the probability of detection was 51% (95% UI: 15%, 82%) for the 720-trap system, 30% (95% UI: 8%, 57%) for the 360-trap system, 16% (95% UI: 4%, 35%) for the 180-trap system, and 14% (95% UI: 3%, 38%) for human surveillance. At the highest incidence rate of approximately 1,000 infections per week, all systems had a mean estimated probability of detection of greater than 78%.

**Table 2:** The probability of detecting local transmission of Zika virus and the total number of Zika virus infections detected by testing *Aedes aegypti* pools collected from CDC sentinel autocidal gravid ovitraps and emergency department patients with two or more Zika virus symptoms.

	Vector surveillance			Human surveillance
Strategy performance	180 SAGO (95% UI)	360 SAGO (95% UI)	720 SAGO (95% UI)	
Weekly probability of detection				
ZIKV incidence of 1 per 100,000 per week	1.8% (0.4%, 4.2%)	3.5% (0.8%, 8.2%)	6.9% (1.6%, 15.7%)	1.5% (0.3%, 4.7%)
ZIKV incidence of 1 per 10,000 per week	16.4% (4.0%, 34.7%)	30.1% (7.9%, 57.4%)	51.2% (15.1%, 81.9%)	14.1% (2.7%, 38.0%)
ZIKV incidence of 1 per 1,000 per week	83.3% (33.6%, 98.6%)	97.2% (56.0%, 99.9%)	99.9% (80.6%, 100%)	78.0% (24.2%, 99.2%)
Total number of ZIKV infections detected per week				
ZIKV incidence of 1 per 100,000 per week	0.02 (0.01, 0.04)	0.04 (0.01, 0.09)	0.07 (0.02, 0.17)	0.02 (0.01, 0.05)
ZIKV incidence of 1 per 10,000 per week	0.18 (0.04, 0.43)	0.36 (0.08, 0.85)	0.72 (0.16, 1.71)	0.15 (0.03, 0.40)
ZIKV incidence of 1 per 1,000 per week	1.79 (0.41, 4.26)	3.58 (0.82, 8.52)	7.17 (1.64, 17.07)	1.51 (0.28, 4.79)

UI, uncertainty interval; SAGO, sentinel autocidal gravid ovitraps

**Fig 2:**
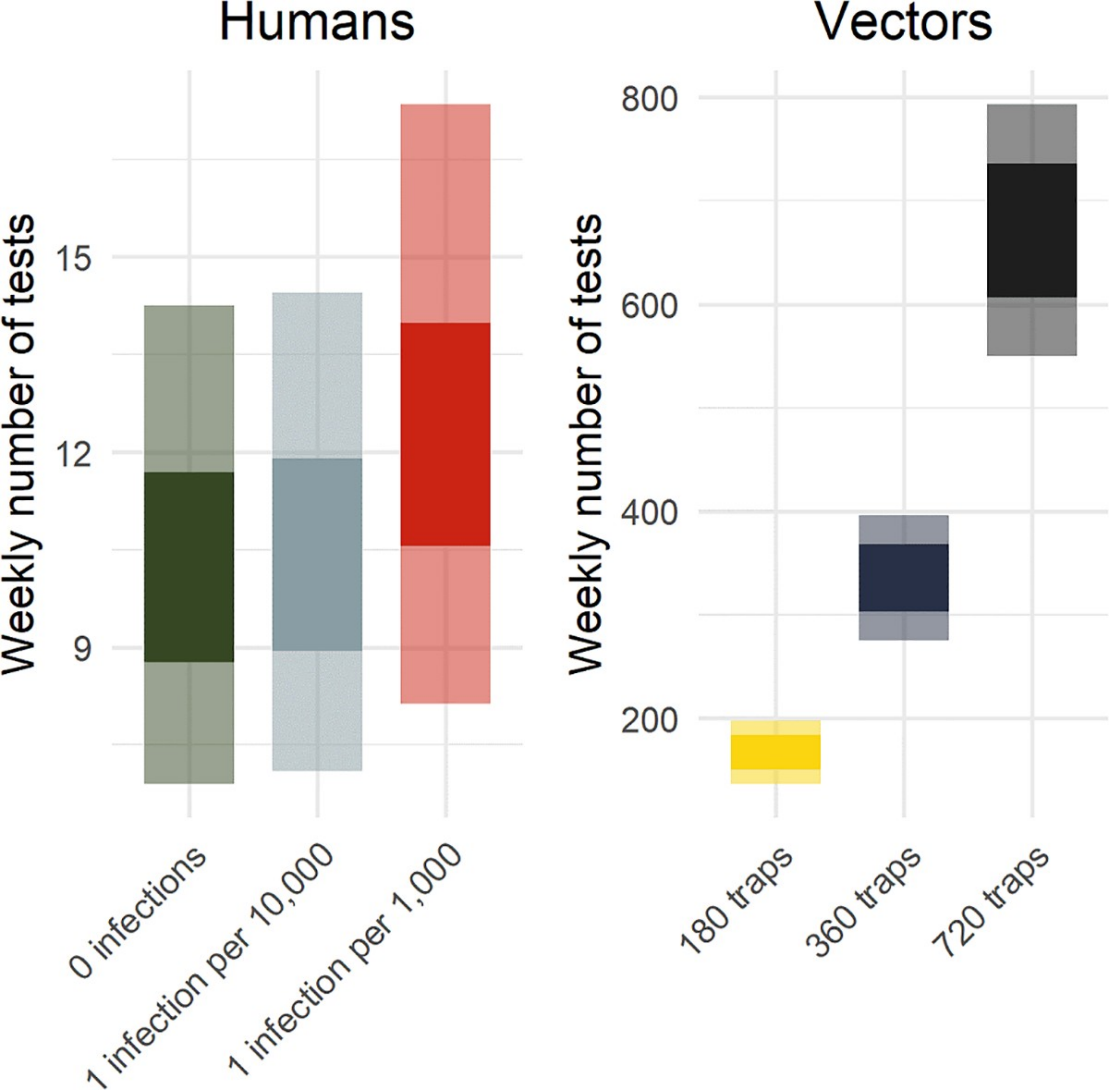
Expected number of tests for vector and human surveillance strategies. This figure shows the 50% uncertainty interval (UI, dark) and 95% UI (light) for the expected number of RT-PCR tests required per week for different surveillance strategies and transmission levels in a population of 100,000 people. The human surveillance strategy indicates the number of tests among emergency department patients with two or more ZIKV symptoms given transmission scenarios of 0 (green), 1 (grey), and 10 (red) infections per 10,000 people per week. The yellow, blue, and black bars are the number of tests required for pools of female *Ae*. *aegypti* for different numbers of traps in the same population.

For the mosquito surveillance systems, higher numbers of traps increased the probability of detection; however, more traps also led to more tests and increased resource needs (Fig 2). In contrast, for human surveillance of patients with ZIKV symptoms, the expected testing resources required were much lower and only increased slightly with higher incidence. For example, with 360 traps, an estimated 335 (95% UI: 275, 396) tests were required per week for vector surveillance. In contrast, approximately 10 (95% UI: 6, 14) tests per week were required for human surveillance with no ZIKV transmission and 12 (95% UI: 8, 17) tests per week with a high transmission scenario of 1,000 infections per week. Both systems had similar positive predictive values, with human surveillance being somewhat higher in low incidence settings (S1 Fig).

**Fig 3:**
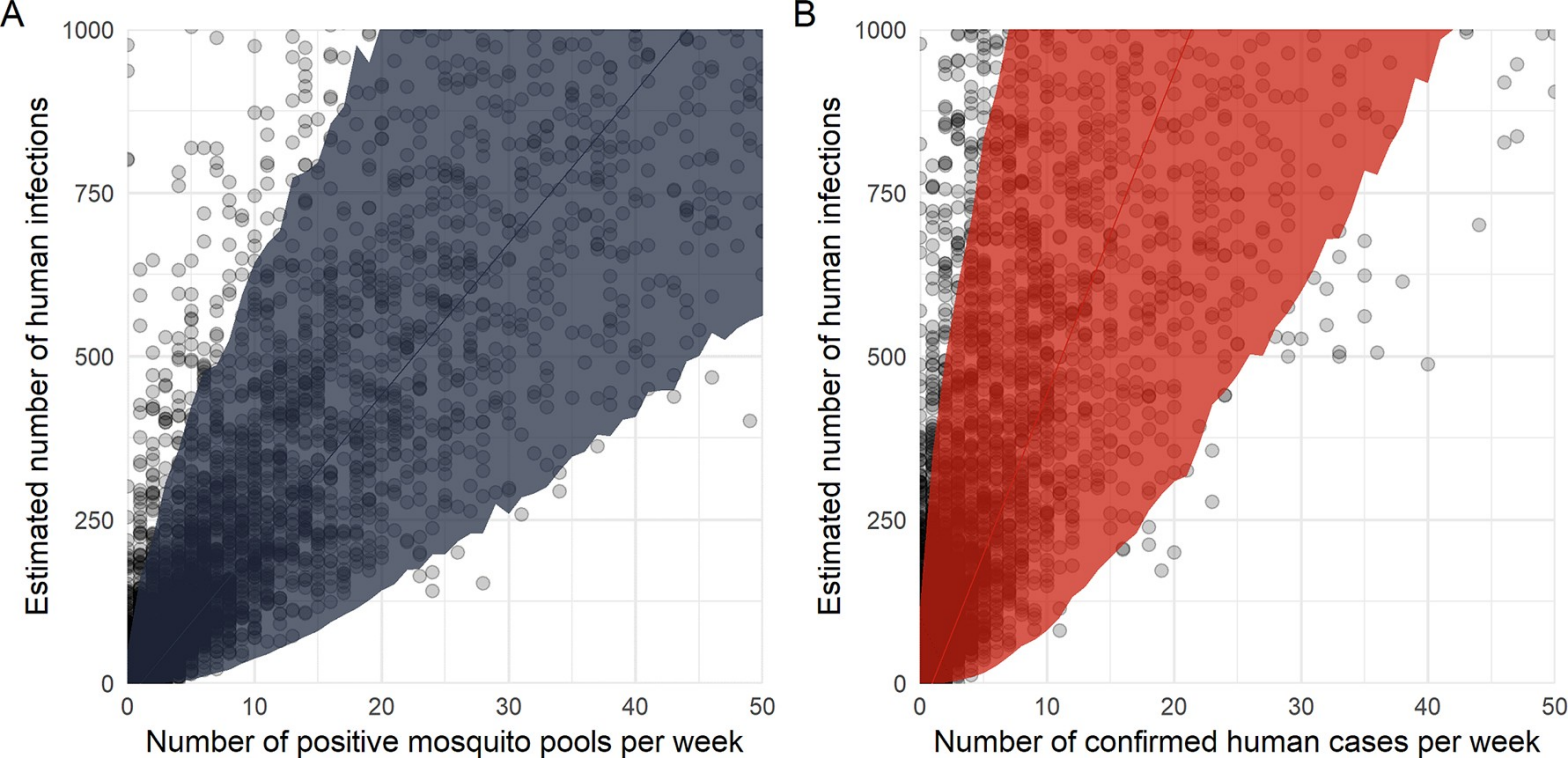
Relationship between the number of simulated Zika virus positive mosquito pools and human infections, and estimated incidence rate of human infections. Panel A shows the estimated incidence of human infections per week by the number of Zika virus positive female *Ae*. *aegypti* pools per week. Panel B is the estimated incidence of human infections per week by the number of infections in emergency department patients with two or more Zika virus symptoms per week. The lines are Bayesian negative binomial regression models fitted to predict the estimated incidence of human infections from the number of positive mosquito pools and the number of humans with two or more symptoms. The bands represent 95% credible intervals.

To identify the relationship between detected infections and incidence, we assessed the relationship between the simulated infection incidence and the observed number of positive pools or human cases in the same simulations (Fig 3). There was high variability across simulations and high uncertainty in the relationship between the number of observed positive pools or confirmed human cases and the number of underlying infections. For each positive mosquito pool, we estimated an additional 23.3 infections (95% Credible Interval (CI): 0.7, 171.1) (Fig 3A). For each confirmed human case detected, we estimated 49.0 additional infections in a population of 100,000 people (95% CI: 0, 334.1) (Fig 3B).

**Fig 4:**
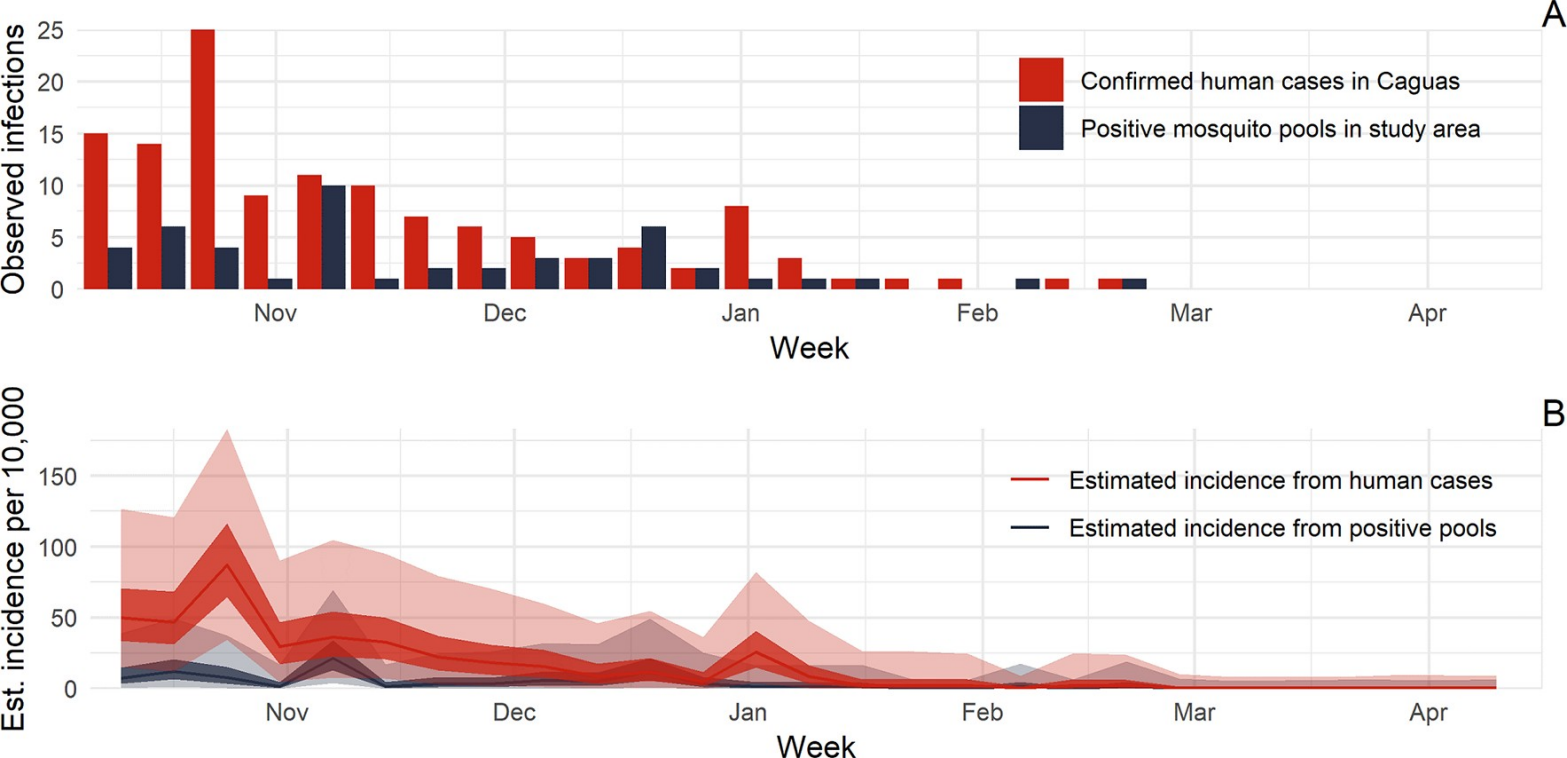
Time series relationship between the actual number of positive mosquito pools and human cases in Caguas, Puerto Rico, and estimated incidence rate of human infections. Panel A shows the actual number of ZIKV positive mosquito pools (navy bars) in the Caguas study area and confirmed human cases (red bars) in Caguas municipality from October 10, 2016, to April 16, 2017. In Panel B, we used the model to estimate the incidence rate of human infections from the number of positive mosquito pools (navy line) and the number of confirmed human cases (red line). The dark bands represent 50% credible intervals (CI), whereas light bands represent 95% CI for the estimated incidence of population infections.

Finally, we compared these simulations to the data reported from Caguas from October 10, 2016, to April 16, 2017. Over this period, there were 127 confirmed human ZIKV cases out of 452 suspect cases detected through passive surveillance (28.1% positive) in the entire municipality of Caguas and 49 ZIKV positive pools of female *Ae*. *aegypti* out of 8,518 pools tested (0.6% positive) in the area of Caguas with vector surveillance (Fig 4A). We reran the simulations using the populations of all of Caguas (130,000) and the study area (80,000) for the human and vector surveillance systems, respectively, to estimate the relationship between the number of infections in populations of these specific sizes and the number of positive mosquito pools or confirmed human cases detected. The relationships were similar to those generated with a uniform population size of 100,000 people for each system; mean expected number of infected humans increasing by 47.6 (95% CI: 0, 341.9) and 19.7 (95% CI: 0.6, 167.4) infections per additional confirmed case or positive pool, respectively.

We used this model to estimate the incidence of infection on a weekly basis using the observed number of confirmed cases and positive pools and calculated the rate of infection given the distinct population sizes (Fig 4B). Although the 95% CIs overlapped for all weeks, the 50% CIs had minimal overlap during late 2016 when incidence in both systems was relatively high. Overall, estimates based on human surveillance were substantially higher in most weeks of 2016 and the incidence rate of the entire study period was 90 (95% CI: 50, 140) infections per 10,000 people based on mosquito pool data in the vector surveillance area versus 500 (95% CI: 340, 680) based on human surveillance data in all of Caguas.

## Discussion

Population-based surveillance and detection of ZIKV is critical to guide timely public health and medical responses to emerging epidemics. Optimal surveillance strategies should be sensitive, specific, and cost-effective [29]. *Aedes aegypti*-transmitted arbovirus surveillance systems have widely focused on testing the human population at risk. In a previous comparison of human-focused ZIKV surveillance approaches, a strategy focusing on patients seeking emergency department care was found to be more effective and efficient than routine testing of pregnant women or blood donors [5]. Virological surveillance of *Aedes* transmitted arboviruses in *Ae*. *aegypti* provides an alternative approach to early detection of virus circulation and monitoring of transmission dynamics during outbreaks that does not depend on human surveillance.

In our simulation models for high transmission scenarios, both vector and human surveillance strategies effectively detected ZIKV. In low transmission scenarios, routine mosquito surveillance with high trap numbers had higher detection probabilities, although the probability of detection was still low (7%). This increased sensitivity also implies that for every confirmed human case there are more infected humans in the population than for every positive mosquito pool. That difference may result from the higher barriers to testing among humans in our model, which included developing two or more symptoms seeking care in an emergency department, and being viremic at the time of testing [5, 30]. Identification of a positive mosquito pool is not influenced by the same contingencies.

In contrast to the simulations, more human cases (127) than positive pools (49) were observed in Caguas, suggesting that human surveillance may be more sensitive than virological surveillance of *Ae*. *aegypti*. Part of this difference is explained by the different population coverage (130,000 for human surveillance and 80,000 for vector surveillance). However, using the model to estimate the infection rate in both populations, we found that the difference was still clear; in almost every week the estimated incidence was higher using human surveillance data. The discrepancy may result from higher risk in the additional 50,000 people covered by human surveillance, but there is no obvious reason for this to be the case. Alternatively, the difference may result from mischaracterization of human surveillance in the simulation model. The model was limited to patients seeking care in an emergency department with two or more ZIKV symptoms, whereas the actual surveillance data likely includes individuals from other health facilities, those with only one ZIKV symptom, patients suspected of having infection for other reasons, and individuals who may have been infected outside of Caguas. These components commonly vary across surveillance systems and locations, and all of these could lead to underestimates of the sensitivity of human surveillance in the simulations.

Overall, the simulations suggested that virological surveillance of *Ae*. *aegypti* as performed in Caguas could potentially provide improved sensitivity to detect virus transmission, but the data from Caguas tell a different story. The observed data confirm that both systems were able to detect transmission in high and low incidence weeks, but they also indicated higher sensitivity for human surveillance. It is also important to consider these approaches in context. The Caguas study was conducted in high and low incidence periods after the peak of the outbreak in July 2016, when clinicians and the local population were already sensitized to ZIKV, which may have increased the number of observed cases. Passive surveillance for arboviral diseases in clinical patients is routine in Puerto Rico [31] and many other locations, so testing of humans may represent little or no additional resource burden. In contrast, mosquito surveillance is less widely implemented and thus may require even more resources, especially for laboratory testing. The resource investment of mosquito-based surveillance is directly related to the sensitivity, which is not necessarily true for human surveillance. In the absence of human surveillance systems or to identify local transmission risk by detecting infected mosquitoes, virological surveillance of *Ae*. *aegypti* may be an effective approach.

An important limitation to this study is that we did not collect data on attendant costs required for virological surveillance in mosquitoes and humans (e.g., traps, field work, laboratory supplies). These resource considerations are not trivial in terms of man hours and laboratory test costs. A second limitation was the considerable uncertainty around key model parameters. We accounted for uncertainty by sampling from parameter distributions, but those distributions were based on data from other locations and therefore do not necessarily reflect the reality of surveillance in Caguas. As described above, this was particularly evident when comparing the simulations to the observed data. We also used a very generic representation of trapping effort, including only the number of traps per person and collections based on the data from Caguas. We therefore ignored all components of spatiotemporal variability in vector populations, trapping effort, and incidence, despite their importance [32, 33].

The simulations suggested some potential gains in sensitivity for virological surveillance of *Ae*. *aegypti*, a finding substantiated by the detection of ZIKV in mosquitoes in Mexico without reported human cases [34]. However, the increased resources needed are likely prohibitive in most circumstances. For example, during the study period there were 8,518 tests on mosquito pools in a subsection of Caguas compared to 452 tests on suspect human cases in all of Caguas. Some of the testing burden may be ameliorated by using “superpools” for mosquito testing [35], but the trapping itself also remains a challenge. Moreover, despite the intensive mosquito trapping and testing in Caguas and the small proportion of ZIKV-infected people who seek care, human surveillance was more sensitive than mosquito surveillance. In communities without health care facilities or in which people do not seek care, virological surveillance in mosquitoes have an important role. Virological surveillance in *Ae*. *aegypti* could also potentially help detect low levels of virus circulation, but only with extensive trapping and testing. In most situations where human surveillance systems already exist, such as in Caguas, human surveillance is likely to be more effective.

## Supporting information

S1 Checklist STROBE checklist. (DOC)

S1 Fig Positive predictive value of testing mosquito pools for Zika virus and emergency department patients with two or more Zika virus symptoms. Panel A describes the positive predictive value (PPV) of a single positive Trioplex Real-time RT-PCR Assay test result on a pool of *Ae*. *aegypti* females. Panel B describes the PPV of a single positive RT-PCR test result on an emergency department patient. The bands represent 50% uncertainty intervals for the PPV of a positive test over a range of possible ZIKV incidences.
(TIF)
