# Supplementary figures and images for: Comparing vector and human surveillance strategies to detect arbovirus transmission: A simulation study for Zika virus detection in Puerto Rico
Source: PLoS Negl Trop Dis. 2019 Dec 26;13(12):e0007988. doi: 10.1371/journal.pntd.0007988 (PMC6948821; doi:10.1371/journal.pntd.0007988)

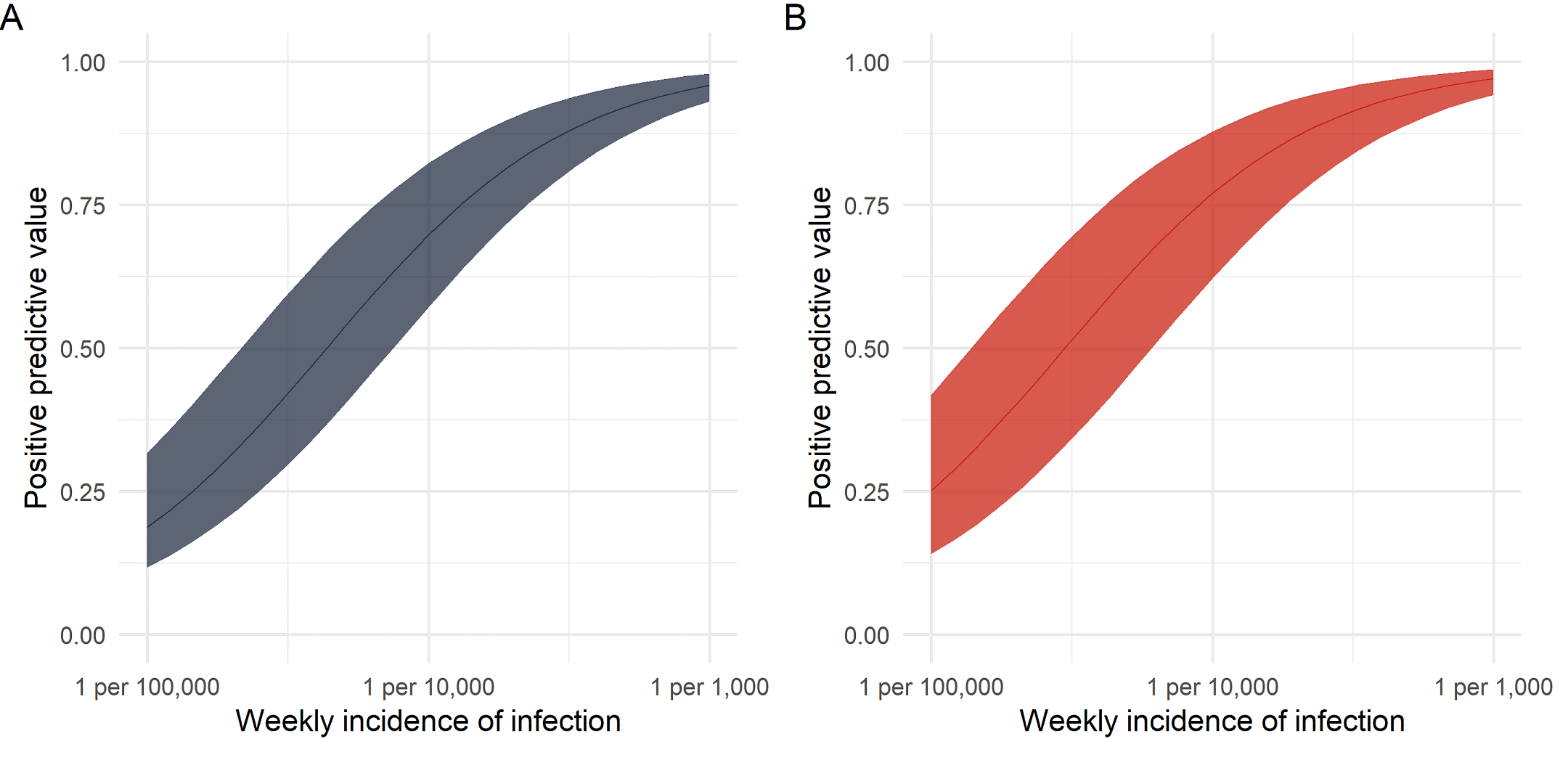

Supplement: S1 Fig — Panel A describes the positive predictive value (PPV) of a single positive Trioplex Real-time RT-PCR Assay test result on a pool of Ae. aegypti females. Panel B describes the PPV of a single positive RT-PCR test result on an emergency department patient. The bands represent 50% uncertainty intervals for the PPV of a positive test over a range of possible ZIKV incidences. (TIF) [file pntd.0007988.s002.tif]
